# Application of Low-Cost MEMS Spectrometers for Forest Topsoil Properties Prediction

**DOI:** 10.3390/s21113927

**Published:** 2021-06-07

**Authors:** Felix Thomas, Rainer Petzold, Carina Becker, Ulrike Werban

**Affiliations:** 1 Helmholtz Centre for Environmental Research—UFZ, Department Monitoring & Exploration Technologies, Permoser Straße 15, 04318 Leipzig, Germany; ulrike.werban@ufz.de; 2 Public Enterprise Sachsenforst, Unit Site Survey, Soil Monitoring and Laboratory, Bonnewitzer Straße 34, 01796 Pirna, Germany; Rainer.Petzold@smul.sachsen.de (R.P.); Carina.Becker@smul.sachsen.de (C.B.)

**Keywords:** forest soil, humus, proximal soil sensing, vis-NIR spectroscopy, MEMS-spectrometer

## Abstract

Increasing temperatures and drought occurrences recently led to soil moisture depletion and increasing tree mortality. In the interest of sustainable forest management, the monitoring of forest soil properties will be of increasing importance in the future. Vis-NIR spectroscopy can be used as fast, non-destructive and cost-efficient method for soil parameter estimations. Microelectromechanical system devices (MEMS) have become available that are suitable for many application fields due to their low cost as well as their small size and weight. We investigated the performance of MEMS spectrometers in the visual and NIR range to estimate forest soil samples total C and N content of Ah and Oh horizons at the lab. The results were compared to a full-range device using PLSR and Cubist regression models at local (2.3 ha, n: Ah = 60, Oh = 50) and regional scale (State of Saxony, Germany, 184,000 km${}^{2}$, n: Ah = 186 and Oh = 176). For each sample, spectral reflectance was collected using MEMS spectrometer in the visual (Hamamatsu C12880MA) and NIR (NeoSpetrac SWS62231) range and using a conventional full range device (Veris Spectrophotometer). Both data sets were split into a calibration (70%) and a validation set (30%) to evaluate prediction power. Models were calibrated for Oh and Ah horizon separately for both data sets. Using the regional data, we also used a combination of both horizons. Our results show that MEMS devices are suitable for C and N prediction of forest topsoil on regional scale. On local scale, only models for the Ah horizon yielded sufficient results. We found moderate and good model results using MEMS devices for Ah horizons at local scale ($R^{2}\geq \;$ 0.71, RPIQ ≥ 2.41) using Cubist regression. At regional scale, we achieved moderate results for C and N content using data from MEMS devices in Oh ($R^{2}\geq \;$ 0.57, RPIQ ≥ 2.42) and Ah horizon ($R^{2}\geq \;$ 0.54, RPIQ ≥2.15). When combining Oh and Ah horizons, we achieved good prediction results using the MEMS sensors and Cubist ($R^{2}\geq \;$ 0.85, RPIQ ≥ 4.69). For the regional data, models using data derived by the Hamamatsu device in the visual range only were least precise. Combining visual and NIR data derived from MEMS spectrometers did in most cases improve the prediction accuracy. We directly compared our results to models based on data from a conventional full range device. Our results showed that the combination of both MEMS devices can compete with models based on full range spectrometers. MEMS approaches reached between 68% and 105% of the corresponding full ranges devices $R^{2}$ values. Local models tended to be more accurate than regional approaches for the Ah horizon. Our results suggest that MEMS spectrometers are suitable for forest soil C and N content estimation. They can contribute to improved monitoring in the future as their small size and weight could make in situ measurements feasible.

## 1. Introduction

The drought events of 2003 and 2010 broke the 500 year record, and the probability of occurrence of extreme heat events is increasing in the future due to global climate change [1]. In 2018, this record was broken again [2], with immediate effects on vegetation growth and further causing soil moisture depletion [3]. As direct consequence, tree mortality increased in many species. As trees are highly vulnerable to other impacts such as insect calamities or fungal attacks after the drought, mortality is likely to go on for years [4].

In Europe, forest areas affected by mortality doubled since 1984, with increasing annual temperature being identified as one of the causes [5]. As a result of the extreme drought stress, the 2018 event caused unprecedented drought-induced tree mortality in many species throughout the region. Moreover, unexpectedly strong drought legacy effects were detected in 2019. This implies that the physiological recovery of trees was impaired after the 2018 drought event, leaving them highly vulnerable to secondary drought impacts. As a consequence, the whole extent of mortality of trees triggered by the 2018 events can not be foreseen yet. In North America, increased rates of forest dieback are reported, linked to drought and rising temperatures [6,7]. Evidence for heat-induced tree mortality was found around the whole globe [8]. Forest ecosystems will be substantially altered by these ongoing effects, which will as well have a strong impact on forest soils. Gessler et al. [9] state that high nutrient availability in soils can increase water use efficiency. Further, organic substance can positively influence water storage capacity in soils [10,11]. Therefore, apart from observation of soil moisture and forest vegetation, there is a clear need for improved soil monitoring to ensure proper detection of changes in soil nutrients availability. Methods of proximal soil sensing can be helpful for fast, non-invasive and cost-effective determination of soil properties [12].

On agricultural sites, numerous studies successfully applied vis-NIR spectroscopy to predict soil properties on field scale, e.g., [13,14,15,16]. There are examples for application of the method on different larger scales as well, e.g., for Australia [17], Europe [18] or Belgium and Luxembourg [19]. Some results proved that accuracy is decreasing with extension of the geographic range the samples were originating from [20]. Other studies also showed that local calibrations result in best prediction performance [21].

Regarding forest ecosystems, ref. [22] used near-infrared spectroscopy to estimate total and exchangeable cations and [23] successfully predicted carbon and nitrogen content. The spectral ranges important for prediction of physical properties lie around 480 and 580 nm in the visual range and around 1400, 1900 and 2200 nm in the NIR-range of the electromagnetic spectrum [24]. Unlike as in agricultural areas, satellites or airplanes can not be used to gain spectral information of forest soils due to the permanent vegetation cover. Using information gained from vegetation as proxy remains difficult and inapplicable. Even most recent approaches show poor results when compared to predictions based on bare soil data [25]. Measurements of forest soils are therefore mostly taken after sample collection under laboratory conditions. Due to the big size and heavy weight of the available devices, successful in situ measurements are scarce.

Recently, low-cost microelectromechanical system (MEMS) spectrometer with reduced spectral range are becoming available. Due to their size and weight, these devices are portable and possibly suitable for in situ measurements. This could reduce field campaign cost and make small-meshed recording of soil properties more feasible.

First studies show that these devices can as well be used for soil properties prediction. Refs. [26,27] report model performances comparable to standard full-range devices. Their applicability for fertilizer recommendations are investigated as well [28]. However, there are so far no investigations evaluating their performance on forest soil humus and topsoil layers. In addition, information about the influence of the size of the study area on model predictions for forests are rare. Investigations comparing local and regional scales were mainly carried out on agricultural soils. Forest soils are different as they have an organic surface layer called O-horizon. It consists of organic matter in different states of decomposition. Within this layer, the Oh horizon consists of humus and amorphous organic material. Below this horizon, the Ah horizons lies as mineral layer, containing less than 30% organic matter. For forests, retained soil samples of the National Forest Soil Inventory (BZE) in Germany are highly suitable as they cover a wide range of parent material and soil types. The objectives of this work are (i) to assess the accuracy of forest humus properties prediction in Oh and Ah horizons based on MEMS-spectrometers using solely the visual, the near-infrared range and a combination of both, (ii) to compare the results to models build using data from full-range devices and (iii) see if and how model accuracy depends on the different spatial scale the samples originate from.

## 2. Methods

### 2.1. Study Area and Sampling Design

In this study, we used data sets collected at two different spatial scales. On one hand, we used retained samples from the periodic BZE in the federal state of Saxony, Germany, supplemented with data from sites representing typical natural areas of Saxony (see [29]). The BZE Saxony sampling was conducted from 2006 to 2014. Samples in this data set originate from all over Saxony (18.400 km${}^{2}$). More information on the methodology of the periodic BZE sampling can be found in [30]. From all 727 available retained samples, we selected a subsample that represents abundant vegetation types and parent materials in Saxony. The focus lay on dominating forest stands. Conditioned Latin hypercube sampling (clhs) was used for samples from spruce and pine stands to get the optimal number of samples. Further, samples from mixed stands, and minima, median and maxima per horizon and humus form were added, resulting in 362 samples. This data set includes samples from organic and mineral horizons, with 176 samples from Oh-horizon (humified plant material) and 186 samples from Ah horizon (mineral layer with less than 30% organic matter). The main parent materials are sand, loess loam, gneiss, granite, shale and phyllite. Cambisols, gley and podsols are the most abundant soil types. This data set is referred to as “BZE Saxony”.

**Figure 1:**
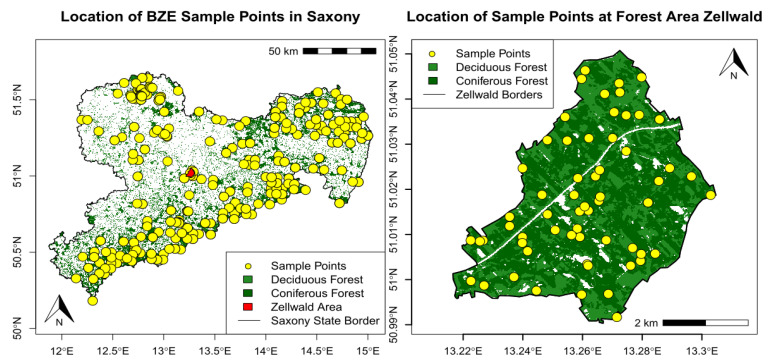
Location of BZE Sample Points in Saxony with Forest Area Zellwald (left), Location of the Sampling Points within the Zellwald Area (right), vegetation data provided by [31].

On the other hand, we collected 110 (50 from Oh and 60 from Ah horizon) further samples for a second data set at forest stand scale in a field campaign that took place in June and July 2019 at the forest site “Zellwald” (2.3 ha), located in Middle Saxony. Parent material is loess loam, dominant soil types are luvisols and stagnic luvisols. For this data, the sampling points were selected using clhs sampling beforehand to ensure data collection at representative points and a good representation of the areas soil properties variability. Therefore, all collected samples were used. The data set is referred to as “Zellwald”. The location of the sampling points in Saxony and at the forest site “Zellwald” can be seen in Figure 1.

The study design described in the sampling standard for the BZE was used as basis for the acquisition [32]. The samples were collected using a satellite sampling scheme with eight satellites per point. In case that, due to the site conditions, one or more satellites could not be probed, it was shifted inwards or outwards the circle to facilitate the collection. At every satellite, soil material from Ah horizon and, if present, from organic Oh-horizon was collected and mixed for each horizon. This procedure results in one sample per horizon per sampling point.

### 2.2. Laboratory Analysis

All chemical analysis was performed by the laboratory of Sachsenforst public enterprise, following german-wide standards for forest soil chemical analysis [33]. Total carbon (C) and nitrogen (N) fractions were determined. For total carbon content the dry combustion method with elementary analysis was applied [34].

Before the spectral analysis was conducted, all samples were dried and sieved with a 2 mm mesh to ensure homogeneous material. To protect volatile compounds of the soil during the drying process, the temperature for the samples was set to 40 ${}^{\circ }$C.

The spectral measurements were acquired following a self-developed protocol, which was created based on literature research and several trial measures. Aims of the protocol were to capture each samples variability, to enable a fail-safe procedure and ensure comparability of the measurements. Per soil sample, two petri dishes were filled with soil material. Each dish was then measured five times, rotating the dish after every measure to increase the acquired variability. Further, this procedure balances the values within the measured area [35]. According to the protocol, the procedure results in ten spectra per sample.

The R language for statistical computing was used for all processing and calculation steps in this study [36].

During the spectral measuring, light scatter can result in effects like baseline shifts or other anomalies. To antagonize these effects, numerous suitable preprocessing techniques exist [37]. First, we smoothed our data using the Savitzky–Golay-Filter. It calculates the sum over a given window, computed as follows in Equation (1):

(1) $$x_{j}*=\frac{1}{N}\sum_{h=-k}^{k}c_{h}x_{j+h}$$

where $x_{j}*$ is the new value, *N* is a normalizing coefficient, *k* is the gap size on each side of *j* and $c_{h}$ are pre-computed coefficients, that depend on the chosen polynomial order and degree [38,39]. We used the third polynomial and a window size of 11.

We further normalized our spectra using standard normal variate (SNV). It is calculated as displayed in Equation (2):

(2) $$SNV_{i}=\frac{x_{i}-\bar{x_{i}}}{s_{i}}$$

where $x_{i}$ is the signal of a sample *i*, $\bar{x_{i}}$ is its mean and $s_{i}$ its standard deviation [37,40]. The above described and applied pre-processing steps were carried out by means of the the R package prospectr [39].

### 2.3. Sensors

We used three different spectrometers in our study: one full-range vis-NIR devices (Veris Technologies Inc., Salina, KS, USA), one MEMS-spectrometer for measuring in the visible range (Hamamatsu Photonics K.K.) and one MEMS-spectrometer capturing the NIR range only (Si-Ware Systems Inc., Menlo Park, CA, USA). An overview of the devices and their technical specifications can be seen in Table 1.

The Veris device uses two internal spectrometers (Ocean Optics USB4000 and Hamamatsu C9914GB). At the beginning and end of the spectra of the Hamamatsu C12880MA device some bands were cut off to match the transition range of the used optical fibre.

Furthermore, for the Veris spectromters bands were cut of at the beginning and end due to a known high amount of noise. Data between the ranges of the two Veris spectrometers (1000 to 1100 nm) were removed. Calibration of the Veris spectrometer was done using four Fluorillon grey standards as external references [41]. The other devices were calibrated by means of a Spektralon plate. In our study, we investigated the performance of each single device and moreover, the data from the Hamamatsu and NeoSpectra device were merged into one vis-NIR data set to evaluate combined performance of the MEMS-spectrometers using the visual and near-infrared range at the same time. This data fusion results in a gap between the ranges of devices from 850 to 1350 nm and therefore still contains less spectral information than the full-range device.

### 2.4. Regression Analysis

For forest soil properties predictions, we selected two regression approaches. First, partial least square regression (PLSR) was applied, which can be seen as widely known standard method to for soil properties prediction using vis-NIR spectral data [42]. It was initially introduced as a solution for multivariate calibration problems using spectroscopy [43]. PLSR constructs a set of linear combinations of the input data by extracting a sequence of derived, orthogonal directions [44]. These so called factors have high variance as well as high correlation with the response variable and are used for the regression instead of the original data. In this way, the amount of data and therefore the computation time are reduced considerably.

As second method from the field of machine learning, we used the Cubist regression model. The technique is based on the M5 algorithm. It constructs tree-based linear models and is suitable for learning tasks with high dimensionality [45]. It is based on a regression tree, where intermediate linear models at each step are the basis of the predictions. By using subsets of the original data with similar attributes, rules for regression are set by selecting the best predictor, which is used as regression variable. The rules are then connected using if/else statements. If a condition is fulfilled, the regression rule for this subset is applied, otherwise, the next rule is probed [46]. The advantage of this approach is the capability of detecting non-linear relationships [47]. Recently, it was successfully applied for soil properties prediction. Ref. [48] used cubist amongst other methods to predict soil organic carbon (SOC), total nitrogen (TN) and pH values and found it to be superior amongst different algorithms. Similar results were reported by [27] when estimating SOC and total C of agricultural soils based on spectra recorded with different devices.

### 2.5. Model Tuning and Validation

Model calibration was done by means of a ten-fold cross validation and independent validation was performed using parts of the data as test set. Accordingly, we split our data into a calibration and validation set using a ratio of 70:30. In this way, 70% of the data is used to calibrate the model. The remaining 30% are held back and used for independent validation of the prediction performance after the regression models are calibrated.

We calibrated models for Oh and Ah horizons for the regional BZE data and local Zellwald data separately. For the regional BZE data, we also carried out an approach using samples from both horizons. This can be reasonable as the separation of the horizons can be difficult during the sampling. In this case, soil samples of the two horizons from the same sampling point were always kept together as spatial dependence between the horizons my otherwise led to overoptimistic results. The accuracy of the selected algorithms for regression analysis can be improved by tuning specific hyperparameters. In case of PLSR, the number of used components has to be optimized. For Cubist regression, we have to select the ideal number of committees and neighbours. To ensure a robust selection of values for the hyperparameters of each approach, we used a 10-fold cross validation during the model calibration. In this procedure, we randomly split the data into ten subsets of the same size. Again, samples from the same point were kept in the same splits. Then, model calibration is performed with nine of the subsets, and the remaining subset is used for validation of the model. This procedure is repeated ten times, until each of the ten subsets was used as validation for the model performance once. By this, in total ten models are built, always using different parts of the training data and thus resulting in ten estimates of the model performance using all the data in the calibration set. The estimated prediction errors of all ten models is then combined [44]. This ten-fold cross validation was repeated several times, using different values for the hyperparameters of the algorithms. The tuning was done by a grid-search. Regarding PLSR, one up to 20 components were used to optimize the results. For Cubist, we selected the ideal combination of hyperparameters while searching between values of 1 and 50 for committees, values for neighbours ranged from zero to nine. The values resulting in the lowest error were then chosen and used to evaluate the performance on the test data set, which consists of data the model has not seen yet. For regression model calibration, log-transformed values of C and N content were used. All values were back-transformed prior to model performance validation. For assessing the models performance on the test data, we used different error measures. To evaluate the deviation of the predicted from the measured values, we used the root mean squared error (RMSE). The RMSE is computed as can be seen in Equation (3):

(3) $$RMSE=\sqrt[{}]{\frac{1}{n}\sum_{i=1}^{n}{\left(y_{i}-x_{i}\right)}^{2}}$$

where $y_{i}$ are the predicted, $x_{i}$ are the observed values.

Apart from error measures based on the deviations of the predictions to the actual observed values, overall model performance was assessed using the coefficient of determination, $R^{2}$, described in Equation (4):

(4) $$R^{2}=1-\frac{\sum {\left(y_{i}-{\hat{y}}_{i}\right)}^{2}}{\sum {\left(y_{i}-\bar{y}\right)}^{2}}$$

where *y* are the observed, $\hat{y}$ are the predicted values and $\bar{y}$ is the mean value of the observed values.

We computed the ratio of performance to interquartile distance in addition. It is calculated as follows in Equation (5) [49]:

(5) $$RPIQ=\frac{Q3_{obs}-Q1_{obs}}{RMSE}$$

where Q1 and Q3 are the first and the third quantile of the observed values.

Chang et al. [50] suggested a system to rank RPD values. We adapted it for RPIQ (transformation was done by multiplying the values with 1.34896 (as the interquartile range of a Gaussian distribution equals 1.34896 × SD)). Model with RPIQ > 2.70 are considered good, models with 1.89 < RPIQ < 2.70 are moderate and the threshold for poor performance is RPIQ < 1.89.

In general, the models accuracy is indicated by low RMSE and high $R^{2}$ and RPIQ values. All calculations were carried out by means of the R language for statistical computing [36] and the caret package for classification and regression training [51].

## 3. Results

### 3.1. Results of Chemical and Spectral Data Analysis

The summary of the results of the chemical analysis of the soil samples from both data sets are shown in Table 2, separated by horizon. The BZE Saxony data consisted of 362 samples in total, with 176 samples from Oh horizons and 186 samples from Ah horizons. In the organic horizon, values for total C content ranged from 8.60 to 49.4%, with a mean of 27.15%. Percentage of nitrogen in the sample lay between 0.32 and 2.08%. Mean value was 1.22%. For the Ah horizon, C content values lay between 0.40 and 17.22, with a mean of 5.12. The mean of the N content was 0.23, with values ranging from 0.02 to 1.06.

Regarding the data set collected in the forest area Zellwald, values for C content in the Oh horizon were measured to be between 18.81 and 41.36 %, with a mean of 33.99 %. The N content in these samples ranged from 0.2 to 2.11 %. The mean was 1.66 %. In the Ah horizon, C content lay between 3.16 and 13.88 with a mean value of 6.43. Measured values for N content ranged from 0.17 to 1.08, mean value was 0.34.

**Figure 2:**
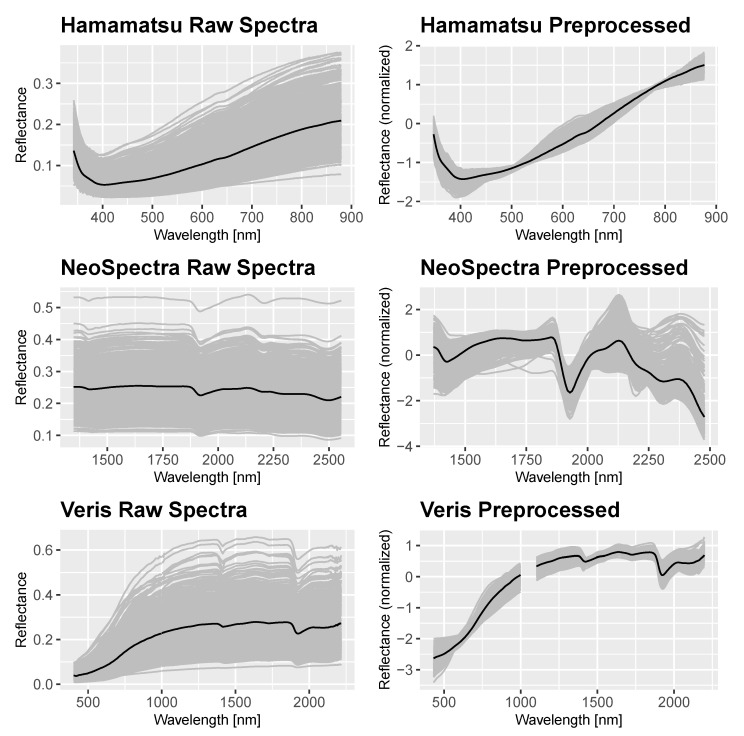
Raw (left) and preprocessed (S-G Filter and SNV, right) spectra of samples from both data sets using the selected devices, the black line represents the mean of all measurements.

Graphs of the spectra obtained with the different devices can be seen in Figure 2. It shows all measured samples (mean of ten single measurements) of both data sets per spectrometer, with the mean of all measurements. The raw spectra is shown on the left, while pre-processed data can be seen on the right side. It clearly can be seen that remarkable trends in all measured spectra were emphasized by the applied pre-processing methods.

The Hamamatsu and Veris spectra both showed the steady ascent through the whole visual range. In the NIR-range, the important reflectance dips around 1400 as well as around 1900 could be detected in both measured spectra using the Veris and Neospectra devices. They are also known as strong water absorption features. The preprocessing accentuated these trend for both devices. In the Neospectra data, the feature around 2200 could be seen as well as it ranges until 2500 nm.

### 3.2. Predicting Total Carbon Content

To evaluate the application of the selected spectrometers for total C prediction of forest topsoil samples, we calibrated models for Oh and Ah horizon using PLSR and Cubist for every spectral data set, once for the BZE data from whole Saxony (regional scale), and once for the forest site Zellwald (local scale). An additional model was calibrated using samples from Oh and Ah horizons combined for the regional scale.

The validation results of the separate models for the Oh and Ah horizons for the regional BZE data set are shown in Table 3. Using the spectral data in the visual range derived by the Hamamatsu device to predict carbon, the results were: RMSE = 8.98%, $R^{2}$ = 0.01 and RPIQ = 1.48 (Oh) and 2.36%, 0.29 and 1.67 (Ah) for the PLSR approach. Using Cubist, RMSE, $R^{2}$ and RPIQ were 7.03%, 0.40 and 2.90 (Oh) and 2.13%, 0.36 and 2.86 (Ah), respectively. When calibrating the models with NIR range data from Neospectra, results for PLSR regression were: RMSE = 6.87%, $R^{2}$ = 0.43, RPIQ = 1.94 (Oh) and RMSE = 1.97%, $R^{2}$ = 0.48 and RPIQ = 2.00 (Ah). The Cubist regression resulted in lower RMSE values of 6.70% and $R^{2}$ and RPIQ of 0.43 and 1.99 for Oh horizons. For the Ah horizon data, model performance was more accurate as well (RMSE = 1.69% and $R^{2}$ = 0.61, RPIQ = 2.34). The combination of Hamamatsu and Neospectra data using both visual and NIR range for regression resulted in RMSE, $R^{2}$ and RPIQ values of: 5.87%, 0.57 and 2.27 (PLSR, Oh), 2.01%, 0.49 and 1.97 (PLSR, Ah), 5.60%, 0.61 and 2.38 (Cubist, Ah) and 1.75%, 0.55 and 2.25 (Cubist, Ah).

**Figure 3:**
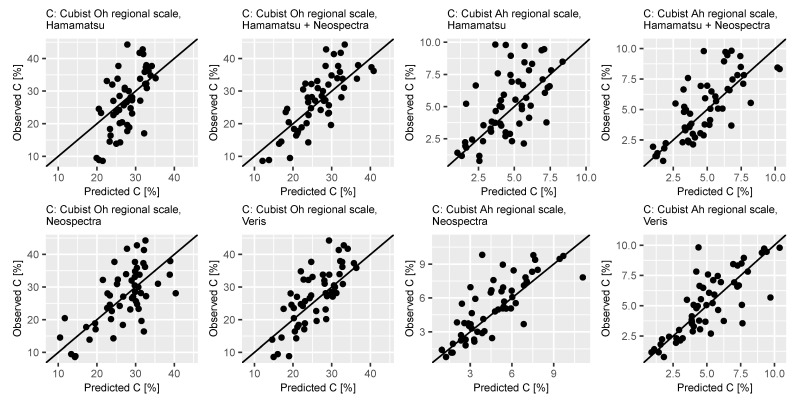
Predicted vs. observed C content values for validation of cubist regression models for Oh and Ah horizon based on spectral data obtained with the selected devices.

**Figure A1:**
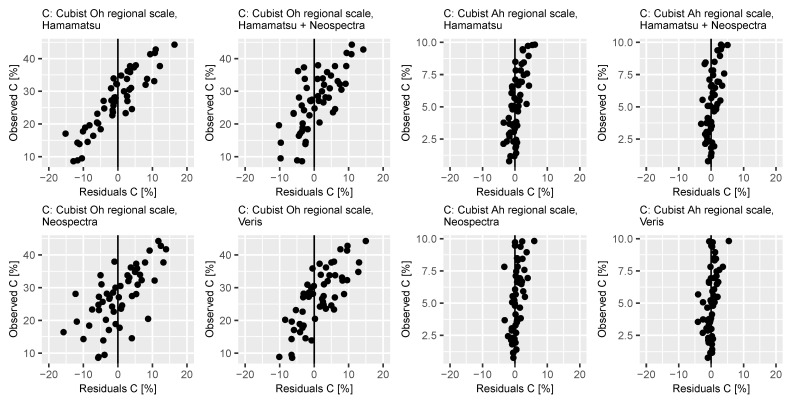
Residuals of C content prediction for validation of separate cubist regression models for Oh and Ah horizon based on spectral data obtained with the selected devices.

Modeling results on basis of the full-range Veris spectrometer with PLSR achieved: RMSE = 6.79%, $R^{2}$ = 0.47, RPIQ = 1.96 (Oh) and RMSE = 1.93%, $R^{2}$ = 0.55 and RPIQ = 2.05 (Ah). The Cubist estimations results were calculated as: RMSE = 5.98%, $R^{2}$ = 0.58 and RPIQ = 2.23 (Oh) and RMSE = 1.59%, $R^{2}$ = 0.62 and RPIQ = 2.48 (Ah), respectively. Therefore, the most accurate predictions of C content were reported using the full-range approaches. The usage of MEMS-spectrometer in combination led to similar predictive performance (Oh: 105%, Ah:89% of the full range models $R^{2}$ values) while the visual range model showed the least precise results (Oh: 69%, Ah: 58% of the full range models $R^{2}$ values). Estimated and observed values of the independent validation sets of the Cubist regression models based on data from all spectrometers and both Oh and Ah horizon are displayed in Figure 3 on the left side. It can be seen that deviations of the predictions from the observed values were not evenly distributed for the models based on the visual range. The other approaches show a better orientation along the x = y line. For the Oh horizon, high values seem to be overestimated while low values tend to be underestimated. The residual plots (see Figure A1 in the Appendix A) underline this observation.

For the regional BZE data, we also calibrated models using a combined approach of both Oh and Ah horizons samples. The results of this approach can be seen in Table 4. Using the visual range to predict C, the results were: RMSE = 6.64%, *R*^2^ = 0.75 and RPIQ = 3.47 (PLSR) and RMSE = 6.36%, $R^{2}$ = 0.76 and RPIQ = 3.63 (Cubist). Using the only the NIR range for model calibration, RMSE, R2 and RPIQ were 6.67%, 0.74 and 3.46 for PLSR and 5.01%, 0.85 and 4.60 for Cubist regression, respectively.

When calibrating the models with data from both visual and NIR ranges combined, results for PLSR were: RMSE = 6.98%, $R^{2}$ = 0.73, RPIQ = 3.30 (PLSR) and RMSE = 5.13%, $R^{2}$ = 0.84 and RPIQ = 4.49 (Cubist). Using the full range spectral data of the Veris device to predict carbon, the results were: RMSE = 5.41%, $R^{2}$ = 0.83 and RPIQ = 4.26 (PLSR) and 4.29%, 0.89 and 5.38 for the Cubist regression approach.

**Figure 4:**
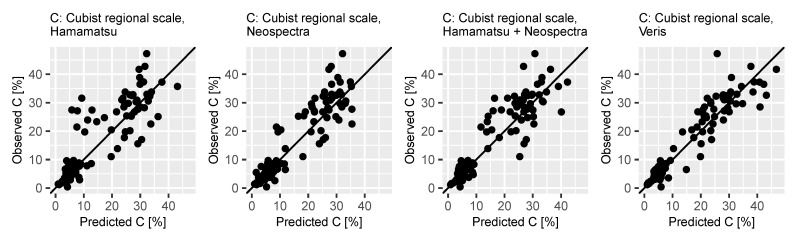
Predicted vs. observed C content for validation of cubist regression models using both Oh and Ah horizon for regional scale (BZE Saxony) based on spectral data obtained with the selected devices.

In this case, the most accurate predictions of C content were reported using the full-range device. The approaches using NIR range alone and MEMS-spectrometer in combination led to similar predictive performance. The model based on the visual range again showed less precise results. Calculated predicted and observed values of the independent validation sets from the BZE data of the Cubist regression models based on data from all spectrometers are displayed in Figure 4. It can be seen that deviations of the predictions from the observed values were generally higher for the samples from Oh-horizons, which were distinguishable from the Ah samples.

For the visual range, it is further notable that, in contrary to the other approaches, the deviations of predicted values were less oriented along the x = y line. Models including data from the NIR area were more precise in this aspect.

The results of the model validation procedure for the Ah horizon of the local Zellwald data set can be seen in Table 5 on the left side.

Using the spectral data in the visual range to predict carbon, the model results were: RMSE = 0.99%, $R^{2}$ = 0.62 and RPIQ = 2.66 for PLSR and 1.08, 0.56 and 2.42 for the Cubist approach. Using NIR spectral data for the models, RMSE, $R^{2}$ and RPIQ were 1.44%, 0.44 and 1.83 (PLSR) and 1.50%, 0.35 and 1.75 (Cubist). When calibrating the models with data from combined visual and NIR range, results for PLSR regression were: RMSE = 1.58%, $R^{2}$ = 0.66, RPIQ = 1.66. For Cubist, model results were RMSE = 0.62%, $R^{2}$ = 0.86 and RPIQ = 4.22. The full-range Veris approach resulted in lower RMSE values of 0.74%, and higher $R^{2}$ and RPIQ of 0.89 and 3.55 for PLSR. For the Cubist model, performance was calculate as RMSE = 0.90%, and $R^{2}$ = 0.86, RPIQ = 2.92.

**Figure 5:**
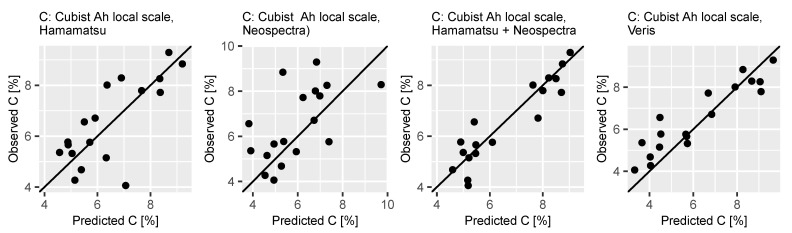
Predicted vs. observed Ah horizon C content values for validation of cubist regression models for local scale (Zellwald) based on spectral data obtained with the selected devices.

Calculated predicted and observed values of the independent validation sets of the Cubist regression models based on data from all spectrometers are displayed in Figure 5. It shows that that the models including data from both visual and NIR range were more accurate, as the points cluster tight around the x = y line. It was not possible to calibrate meaningful models based on the Oh horizon only (data not shown).

### 3.3. Predicting Total Nitrogen Content

In a second step, the performance of the different devices for total nitrogen content prediction was assessed for Oh and Ah horizon for both data sets of the study using PLSR and Cubist.

The results of the separate models of N prediction for the Oh and Ah horizons of the regional BZE data set are shown in the lower part of Table 3. Regressions for Oh horizon based on spectral data in the visual range were more precise using Cubist, with values of RMSE = 0.36%, $R^{2}$ = 0.23 and RPIQ = 1.86. For models based on the Ah samples, performance was less precise.

**Figure A2:**
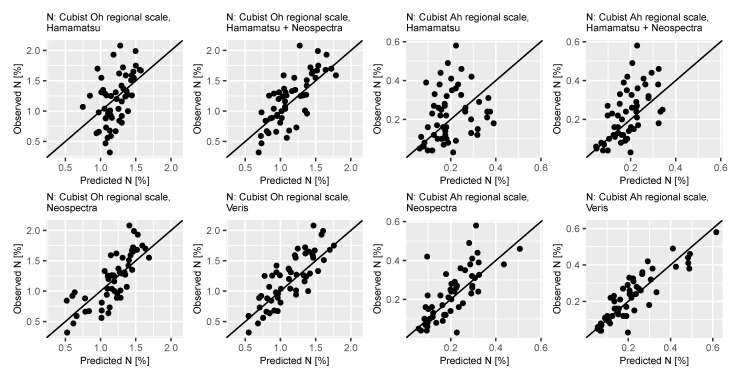
Predicted vs. observed N content values for validation of cubist regression models for Oh and Ah horizon based on spectral data obtained with the selected devices.

For NIR range, Cubist model results were more precise with RMSE = 0.26%, $R^{2}$ = 0.61 and RPIQ = 2.56 for the organic Oh horizon. For the Ah horizon models, we achieved very similiar results for both approaches. Regarding the models for combined data set covering visual and NIR range, Oh results were: RMSE = 0.29%, $R^{2}$ = 0.51, RPIQ = 2.28 (PLSR) and RMSE = 0.27%, $R^{2}$ = 0.57, RPIQ = 2.42 when using the Cubist regression approach. PLSR outperformed Cubist when predicting total N content of the Ah soil samples Additionally, we used a full-range device for model calibration. The models based on Oh samples with PLSR resulted in RMSE = 0.30%, $R^{2}$ = 0.48, RPIQ = 2.42. The Cubist models were more precise with RMSE = 0.25%, $R^{2}$ = 0.66, RPIQ = 2.64. Using Ah samples the Cubist model again resulted in higher accuracy with RMSE = 0.06%, $R^{2}$ = 0.78 and RPIQ = 3.08. In total, the full range device resulted in best prediction performance for total N estimation. Predicted and observed total N content values for models based on data from both horizons and all used spectrometers for the regional BZE data are shown in Figure A2. Largest deviance in the predictions can clearly be seen for the models based on the visual range. Results for the other approaches were more precise, as they were distributed closer and more along to the x = y line. Further, lower values tended to be overestimated, while higher ones were underestimated. This observation was stronger for the models of the Oh horizon.

**Figure A3:**
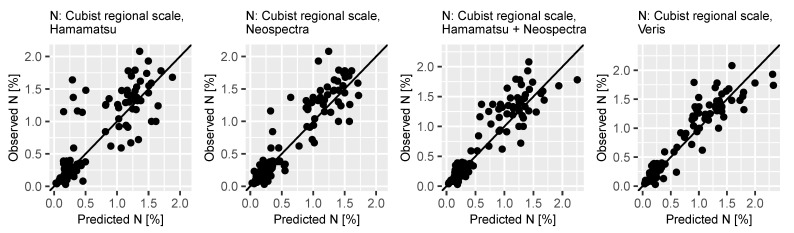
Predicted vs. observed N content for validation of cubist regression models using both Oh and Ah horizon for regional scale (BZE Saxony) based on spectral data obtained with the selected devices.

In addition, we also calibrated models using a combined approach of both Oh and Ah horizons samples for the regional BZE data. The results of the calculations can be seen in Table 4. In this case, the models calibrated based on the visual range to predict N content achieved similar accuracy for both algorithms. Using the NIR range only, model accuracy increased. RMSE, $R^{2}$ and RPIQ were 0.35%, 0.67 and 3.08 for PLSR and 0.24%, 0.85 and 4.51 for Cubist regression, respectively. The results of the combined devices procedure resulted in similar results. PLSR achieved RMSE = 0.34%, $R^{2}$ = 0.71 and RPIQ = 3.17. The usage of Cubist regression resulted in RMSE = 0.24%, $R^{2}$ = 0.85 and RPIQ = 4.57. Models calibrated using data derived from the full-range device yielded best accuracy. The PLSR model resulted in RMSE = 0.29%, $R^{2}$ = 0.79 and RPIQ = 3.80, the Cubist approach in RMSE = 0.21%, $R^{2}$ = 0.88 and RPIQ = 5.24. The predicted and observed values of the Cubist model combing both horizons of the regional BZE data can be seen in Figure A3. The largest inaccuracies in the predictions could be observed for the models based on the visual range. The other approaches were more accurate, indicated by tighter point clouds and a better orientation along the x = y line.

We observed an equivalent prediction power of the combined MEMS-spectrometer approach compared to the Veris device.

**Figure A4:**
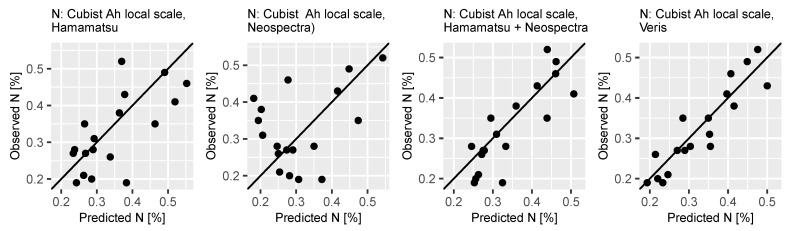
Predicted vs. observed Ah horizon N content for validation of cubist regression models for local scale (Zellwald) based on spectral data obtained with the selected devices.

The results of the independent validation procedure for the Ah horizon of local Zellwald data, separated by algorithm and sensor, can as well be seen in Table 5. The error measures for N content are on the right side. Regression based on spectral data in the visual range was more precise using Cubist and resulted in values of RMSE = 0.08%, $R^{2}$ = 0.45 and RPIQ = 1.67. Regarding the NIR range, model performance increased and was more accurate using PLSR, with RMSE = 0.07%, $R^{2}$ = 0.67 and RPIQ = 2.15 for PLSR. Using Cubist, models were less precise. Using the combination of visual and NIR data, the local model for Zellwald resulted in in RMSE values of 0.09% (PLSR) and 0.06% (Cubist), $R^{2}$ values ranged from 0.58 (PLSR) to 0.71 (Cubist). RPIQ was calculated as 1.54 for PLSR and 2.41 for Cubist. For the full range device data, prediction accuracy was the most precise. PLSR results were: RMSE = 0.06%, $R^{2}$ = 0.87, RPIQ = 2.19. For the Cubist regression, RMSE was 0.04%, R2 was 0.84 and RPIQ = 3.37. Predicted and observed total Ah horizon N content values for models based on local Zellwald data from all used spectrometers are shown in Figure A4. Smallest deviance in the predictions can clearly be seen for the models based on the combined and full rage approaches. As for the C predictions, no suitable models could be calibrated for the Oh horizon of the local Zellwald data.

## 4. Discussion

### 4.1. Feasibility of MEMS-Spectrometer for Forest Soil C and N Content Estimation

This study aimed to investigate the feasibility of using MEMS-spectrometer for forest humus and topsoil total C and N content prediction. We used spectral data measured with MEMS-devices in the visual and NIR range, and a combination of both in addition. Models were calibrated separately for Oh and Ah horizon. Using the regional BZE data, we also modelled C and N content in an approach using both horizons. As Cubist regression showed higher prediction power, the discussion about feasibility and the comparison to conventional devices is based on these results. Models calibrated for Oh samples with the regional BZE data set showed comparable results. Again, the combination of the MEMS devices yielded moderate accuracy for both total C and N content prediction (Cubist: RPIQ ≥ 2.38, $R^{2}$≥ 0.57). The solely usage of the visual range was least precise and it was not possible to establish meaningful models. In the Ah horizon however, the NIR range device had better predictions, reaching the threshold for moderate correlations (Cubist: RPIQ ≥ 2.15, $R^{2}\geq \;$ 0.54). The combination of both devices performed slightly less precise, the Hamamatsu alone was again insufficient (RPIQ < 1.89). In addition, we calibrated models for the regional BZE data using data from both Oh and Ah horizon. In this case, the Neospectra and the combined MEMS devices yielded very similar and good model results (Cubist: RPIQ ≥ 4.49, $R^{2}$≥ 0.84). The predictions based on the visual range showed less accuracy. Even though the model reached the proposed threshold of RPIQ = 2.7, visual assessment revealed high uncertainties within the investigated horizons for both properties. In the Ah horizon of the local Zellwald data, Cubist models using data both MEMS-devices resulted in moderate prediction accuracy for both properties (RPIQ ≥ 2.41, $R^{2}$≥ 0.71). Results for the solely usage of the visual range were moderate only for C content. The estimations based on NIR range only were less precise. Therefore, especially a combination of MEMS devices seemed to be suitable for forest soil C and N content estimation. The solely usage of the visual range however was not sufficient. Regarding the Oh horizon, no meaningful model could be calibrated. One reason for this could be the small sample size.

The similar results of C and N content predictions lead to the question, if soil N content is measured directly, or through auto-correlation with C content. In contrast, other studies presented results indicating that N content is measured independently from C content due to successful prediction of total N and C:N ratios (e.g., [52]).

Further, the results achieved using MEMS devices were to be compared to regression models built on spectral data obtained with a conventional full-range spectrometer. These results were therefore used as benchmark to see if the MEMS devices could reach the predictive performance of the full range device. In most cases the Veris full-range data yielded the best results, with highest $R^{2}$ and RPIQ values and lowest RMSE. However, depending on horizon and scale, it was possible to achieve results that could compete with the models based on the full-range device. Regarding the modelling of Oh samples of the regional BZE data, the Cubist model of combined MEMS approach even outperformed the Veris device for C content (Combined: $R^{2}$ = 0.61, RPIQ = 2.38, Veris: $R^{2}$ = 0.58, RPIQ = 2.23) and therefore achieved 105% of the conventional device. When used alone, the visual range approach had 69% and the NIR range approach 74% of the full range $R^{2}$ value. For N content, the models based on the full range data yielded better results, but the combined MEMS approach could still compete (Combined: $R^{2}$ = 0.57, RPIQ = 2.42, Veris: $R^{2}$ = 0.66, RPIQ = 2.64). In this case, 86% of the full range performance were reached. The models based on NIR spectra solely were more precise ($R^{2}$ = 0.61, RPIQ = 2.56, equals 92%). For Ah horizon, Cubist models based on the NIR range only were the most competitive for C and N content (Neospectra: $R^{2}\geq \;$ 0.54, RPIQ ≥ 2.15, Veris: $R^{2}\geq$ 0.62, RPIQ ≥ 2.48). This equals 98% of the $R^{2}$ values of the Veris device for C content and 69% for N content. The combined approach reached 89% for C and 56% for N content. In general, the NIR range and combined MEMS data devices as well as the full range approach resulted in model performance classified as moderate. Further, we modelled C and N content using samples from both horizons of the regional BZE data set. Similar to the results discussed above, in this approach the Cubist models based on NIR range and the combined MEMS data were able to compete with the full range device for both investigated properties (combined: $R^{2}\geq \;$ 0.85, RPIQ ≥ 4.49, Veris: $R^{2}\geq$ 0.88, RPIQ ≥ 5.24). Therefore, more than 95% of the Veris $R^{2}$ values were reached using MEMS devices, and the calibrated models can be classified as good. However, it is important to mention that the increase of the data range resulting from the usage of both Oh and Ah horizons also led to increased values for the selected error measures. For the Ah horizon of the local approach in the Zellwald forest, the data from combined MEMS devices resulted in comparable accuracy when using Cubist for C (Combined: $R^{2}$ = 0.86, RPIQ = 4.22, Veris: $R^{2}$ = 0.86, RPIQ = 2.92) and N content (Combined: $R^{2}$ = 0.71, RPIQ = 2.41, Veris: $R^{2}$ = 0.84, RPIQ = 3.37). The MEMS as well as the conventional approach yielded moderate to good model predictions. In direct comparison of the $R^{2}$ values of the Cubist models, the combined MEMS approach reached 100% of the full range performance for C content and 85% for the N content.

Our results indicated that the MEMS spectrometers can generally be used to substitute common full-range devices. For the investigated data sets and properties, especially the combination of visual and NIR range data measured with MEMS devices showed predictive performance comparable with the full range device. In some cases, estimations even outperformed the conventional approach.

The two data sets used in this study allowed to investigate the impact of the scale on the model prediction accuracy of the Ah horizon. The results showed that the C content predictions calculated for the local Zellwald forest based on the combined MEMS and the full range devices data were more accurate than the ones based on the BZE data set for both algorithms. Exception is the NIR range model, which performed better on the regional data. The difference in accuracy is bigger for the MEMS-devices than for the full-range spectrometer. Similar observations could be made for the N content estimations. Again, the local results for the combined and full range data models were more precise for both properties. In this case, only the NIR range Cubist results represented an exception. One reason for the differences in the results could be the greater variability in the BZE data set due to the bigger geographical range. Further, data acquisition took place over several years and was therefore carried out by different persons. In addition, there was a longer time span between chemical and spectral measurements. In contrast, the data collection for the Zellwald area was conducted within a short amount of time (approximately one month), all samples were taken by the same samplers and chemical as well as spectral measurements were taken directly in the weeks after the sampling was finished.

### 4.2. Comparison to Other Studies

Few studies investigated the usage of MEMS-devices for soil properties prediction so far. Their applications were using only soil samples originating from agricultural areas. In Australia, e.g., [27] evaluated the prediction power of different low-cost NIR spectrometer for organic as well as total carbon. Using the Neospectra device, they reported $R^{2}$ values of 0.78 (Cubist) and 0.73 (PLSR) for SOC. For total C content, their prediction metrics descended with $R^{2}$ = 0.7 for Cubist and $R^{2}$ = 0.55 when using PLSR. The models were therefore more precise than our results separated by horizon and less precise than our combined models. Another study from Australia investigated the Neospectra for total C content amongst other properties as well [26]. Soil material from 0 to 100 cm depth and a Cubist regression model were used. They achieved $R^{2}$ = 0.74, which is about the same accuracy we calculated using PLSR and combined horizons. However, our estimations based on separate horizons were less precise on local and regional scale. The discrepancies regarding prediction accuracy could lie in the substantial differences in the study setup. All studies investigated agricultural soils, where no humus layer is present. Further, different depth ranges were investigated and the study areas were covering much bigger scales than our study. In a study with focus on precision fertilization in Indonesia [28] reported $R^{2}$ = 0.57 for SOC and $R^{2}$ = 0.52 for total N content, which is within the same range as our regional Ah results. In this case, samples from 0 to 25 cm depth were used.

There were examples of studies using the visible range as well. Ref. [53] estimated SOC using spectral data corresponding to red, green and blue light at field scale, reporting $R^{2}$ = 0.78 using the random forest algorithm. This accuracy equals our regional results based on combined horizons. However, our results based on the visual range were less precise for separated horizons.

Ref. [54] investigated predictions using artificial spectral data, concluding that the range covering 350–975 nm increased inaccuracy when compared to approaches based on, or including NIR range. Ref. [14] found no prediction accuracy difference using vis and NIR range separately to estimate SOC ($R^{2}$ = 0.6 in both cases). This study was conducted on a local approach with sample material from 0 to 20 cm depth originating from an agricultural field in Australia. In our local approach, we reached similar results using the visual range for Ah horizon. However, we found different accuracies between visual and NIR range in the combined horizons approach.

For the differences in prediction accuracy between the local and the regional data set, opposed findings were reported. Ref. [55] investigated the scalability of soil total carbon prediction models using data from different fields individually and in a combined approach. They found the pooled data set and therefore the bigger scale to be more accurate than the single fields. On the other side, Ref. [56] found small scales model calibrations to be more accurate than large libraries when investigating SOC at field scale. Similar observations were made by [20], who reported decreasing prediction accuracy with extension of the geographic range. In our case, the smaller scale also yielded better results. Possible reasons for this observations were suggested, e.g., by [42], who states that model accuracy decreases when containing samples from diverse geographic origins and that the usage of large diverse spectral libraries is therefore more difficult. Ref. [57] reported correlations between the model errors and the standard deviations of the predicted soil properties. It seems to us that the underlying correlations are still not sufficiently unterstood, and that more research including up- and downscaling of models calibrated for forest soils should be done.

### 4.3. Relevance for Forest Soil Monitoring

Based on the good prediction accuracy we achieved under laboratory conditions, we suggest that future in situ measurements are feasible with the investigated MEMS devices due to their small size and weight. This way, obtained data could be used solely or to supplement remote sensing approaches that lack applicability due to the vegetation cover. Further investigations are especially recommended for local forest stands and sites. On agricultural areas, first application of in situ measurements were already conducted [58]. Regarding in situ measurements, it is important to keep the possibility of wet conditions in mind. The influence of water content on spectral data was as well already subject of investigations in agricultural fields [59]. These effects should also be investigated for forest humus and topsoil in order to get reliable in-situ measurements. Another possibility that could be probed is to test a short drying of the soil surface directly before measuring. This way, the impact of soil moisture could possibly be reduced. Another factor that could influence in-situ measurements is the unsieved soil material.

The predictions could, e.g., be used to monitor long term processes. Over the last decades, steadily increasing carbon stocks were reported in forest soils, e.g., in western Europe where an increase of 65% is estimated between 1990 and 2050 [60]. Similar observations were made for China [61]. Such effects could be monitored more easily and with higher spatial resolution by data collection using MEMS devices, as needed efforts can be remarkably reduced.

## 5. Conclusions

In summary, this study demonstrates that low-cost MEMS spectrometers can be successfully used to predict forest humus total C and N content. Models based on spectral data obtained with MEMS are can compete models built with data from full range spectrometer when combining visual (340–850 nm) and NIR (1350–2550 nm) range data. We achieved moderate prediction accuracy for local Ah horizons and regional Oh and Ah horizon samples with data from MEMS and full-range devices. Our results showed that the the performance decreased when using only the visual range, and that it is difficult to establish sufficient models to predict forest total C and N content in this case. Results for Ah horizons were more precise for the local Zellwald area when compared to models based on data from whole Saxony. We conclude that the investigated devices could be suitable for spectral in situ measurements of forest soils due to their small size and weight. The actual application of low-cost MEMS spectrometers in in situ measurements at forest soils should be investigated. Their usage could reduce expenditure of time and costs and of spectral data acquisition and could therefore contribute to build larger spectral libraries of forest soil that can be used for forest soil C and N content monitoring purposes.

## Figures and Tables

**Table 1:** Overview of technical specifications for the selected devices.

Device	Veris Spectrophotometer	NeoSpec SWS62231	Hamamatsu C12880MA
Wavelength	400–2220 nm	1350–2550 nm	340–850 nm
Spectral Resolution	6 nm @ 350–1000 nm, 5 nm @ 1100–2220 nm	16 nm	15 nm
SNR	300:1 @ 400–1000 nm, NA @ 1100–2220 nm	2000:1	NA
Wavelength reproducibility	NA @ 350–1000 nm, ±0.4 @ 1100–2000 nm	±0.15 nm	±0.5 nm
Weight	24 kg	17 g	5 g

**Table 2:** Descriptive statistics for C and N content of Oh and Ah horizons for soil samples from both data sets.

Data Set	Horizon	Parameter	N	Mean	St. Dev.	Min	Pctl (25)	Pctl (75)	Max
BZE Saxony	Oh	C [%]	176	27.15	8.01	8.60	21.73	32.53	49.43
		N [%]	176	1.22	0.38	0.32	0.96	1.53	2.08
	Ah	C [%]	186	5.12	2.77	0.40	2.88	6.99	17.22
		N [%]	186	0.23	0.15	0.02	0.11	0.31	1.06
Zellwald	Oh	C [%]	50	33.99	5.03	18.81	32.11	37.37	41.36
		N [%]	50	1.66	0.20	1.04	1.53	1.79	2.11
	Ah	C [%]	60	6.43	2.14	3.16	4.86	7.81	13.88
		N [%]	60	0.34	0.14	0.17	0.25	0.41	1.08

**Table 3:** Model validation results for total carbon and nitrogen values from Oh (left) and Ah (right) horizon separately using PLSR and Cubist and spectral data from different devices for the regional BZE data set.

			Oh Horizon			Ah Horizon		
Parameter	Algorithm	Sensor	RMSE %	$R^{2}$	RPIQ	RMSE %	$R^{2}$	RPIQ
C		Hamamatsu	8.98	0.01	1.48	2.36	0.29	1.67
	PLSR	Neospectra	6.87	0.43	1.94	1.97	0.48	2
		Hamamatsu + Neospectra	5.87	0.57	2.27	2.01	0.49	1.97
		Veris	6.79	0.47	1.96	1.93	0.55	2.05
		Hamamatsu	7.03	0.40	1.90	2.13	0.36	1.86
	Cubist	Neospectra	6.70	0.43	1.99	1.69	0.61	2.34
		Hamamatsu + Neospectra	5.60	0.61	2.38	1.75	0.55	2.25
		Veris	5.98	0.58	2.23	1.59	0.62	2.48
N		Hamamatsu	0.38	0.18	1.75	0.13	0.17	1.55
	PLSR	Neospectra	0.36	0.31	1.84	0.09	0.55	2.15
		Hamamatsu + Neospectra	0.29	0.51	2.28	0.10	0.52	2.05
		Veris	0.30	0.48	2.21	0.13	0.66	1.48
		Hamamatsu	0.36	0.23	1.83	0.14	0.04	1.37
	Cubist	Neospectra	0.26	0.61	2.56	0.09	0.54	2.15
		Hamamatsu + Neospectra	0.27	0.57	2.42	0.11	0.33	1.74
		Veris	0.25	0.66	2.64	0.06	0.78	3.08

**Table 4:** Model validation results for total carbon and nitrogen using combined horizons (Oh and Ah) for calibration, PLSR and Cubist and spectral data from different devices for the regional BZE data set.

Parameter		C			N		
Algorithm	Sensor	RMSE %	$R^{2}$	RPIQ	RMSE %	$R^{2}$	RPIQ
PLSR	Hamamatsu	6.64	0.75	3.47	0.33	0.72	3.26
	Neospectra	6.67	0.74	3.46	0.35	0.67	3.08
	Hamamatsu + Neospectra	6.98	0.73	3.30	0.34	0.71	3.17
	Veris	5.41	0.83	4.26	0.29	0.79	3.80
Cubist	Hamamatsu	6.36	0.76	3.63	0.33	0.72	3.34
	Neospectra	5.01	0.85	4.60	0.24	0.85	4.51
	Hamamatsu + Neospec	5.13	0.84	4.49	0.24	0.85	4.57
	Veris	4.29	0.89	5.38	0.21	0.88	5.24

**Table 5:** Model validation results for total carbon (left) and nitrogen (right) values from Ah horizon using PLSR and Cubist and spectral data from different devices for the local Zellwald data set.

Parameter		C			N		
Algorithm	Sensor	RMSE %	$R^{2}$	RPIQ	RMSE %	$R^{2}$	RPIQ
PLSR	Hamamatsu	0.99	0.62	2.66	0.10	0.24	1.46
	Neospectra	1.44	0.44	1.83	0.07	0.67	2.15
	Hamamatsu + Neospectra	1.58	0.66	1.66	0.09	0.58	1.54
	Veris	0.74	0.89	3.55	0.06	0.87	2.19
Cubist	Hamamatsu	1.08	0.56	2.42	0.08	0.45	1.67
	Neospectra	1.50	0.35	1.75	0.11	0.15	1.23
	Hamamatsu + Neospectra	0.62	0.86	4.22	0.06	0.71	2.41
	Veris	0.90	0.86	2.92	0.04	0.84	3.37

## Data Availability

The regional data sets from this study are available through the PANGAEA open access repository under https://doi.org/10.1594/PANGAEA.921689 (accessed on 1 February 2021).

## Abbreviations

The following abbreviations are used in this manuscript:

NIR	near-infrared range of the electromagnetic spectrum
vis	visual range of the electromagnetic spectrum
MEMS	microelectro mechanical system
BZE	National Forest Soil Inventory
SOC	Soil organic carbon
clhs	Conditioned Latin hypercube sampling
SG	Savitzky - Golay Filter
SNV	Standard Normal Variate
PLSR	Partial Least Square Regression
RPD	Ratio of Performance to Deviation
